# Inhibitory Effects of Phellodendri Cortex Against Airway Inflammation and Hyperresponsiveness in Ovalbumin-Induced Murine Asthma Model

**DOI:** 10.3390/molecules30081795

**Published:** 2025-04-16

**Authors:** Seong-Kyeom Kim, Ji-Won Do, Seong-Kyun Lee, Jae-Ho Park, Ju-Hyoung Kim, Heung-Bin Lim

**Affiliations:** 1 Bureau of Research & Development, Chungcheongbuk-do Agricultural Research and Extension Services, Cheongju 28130, Republic of Korea; skkim0306@korea.kr (S.-K.K.); dojiwon109@korea.kr (J.-W.D.); lepimoth@korea.kr (S.-K.L.); parkjaeho@korea.kr (J.-H.P.); apox9122@korea.kr (J.-H.K.); 2 Department of Industrial Plant Science & Technology, Chungbuk National University, Cheongju 28644, Republic of Korea

**Keywords:** phellodendri cortex, alkaloid, asthma, airway inflammation, airway hypersensitivity

## Abstract

Phellodendri cortex (PC), the dried trunk bark of *Phellodendron amurense* RUPR, has traditionally been used to treat patients who suffer from gastroenteritis, abdominal pain or diarrhea. Its major bioactive compounds include alkaloids and limonin, and many physiological activities including anti-microbial, anti-ulcer and anti-cancer as well as anti-inflammation have been reported. Although PC is an effective anti-inflammatory natural substance that inhibits the inflammatory response, its effect on allergic asthma has not yet been investigated. The aim of this study was to evaluate the anti-asthmatic effects of PC in an ovalbumin (OVA)-induced murine model of asthma. As a result, PC inhibited airway eosinophil accumulation, the influx of inflammatory cells, airway hyperresponsiveness (AHR), production of Th2 cytokines (IL-4, IL-5 and IL-13) and tumor necrosis factor-α (TNF-α) in the bronchoalveolar lavage fluid and/or lung, as well as OVA-specific immunoglobulin E (IgE) in the serum. Furthermore, PC suppressed the gene expression of IL-4, IL-5, IL-13, TARC and CCR3, and attenuated unique histological changes that are associated with airway inflammatory reactions including the infiltration of various inflammatory cells, collagen deposition and goblet cell hyperplasia in lung tissues. These results indicate that PC may have preventive and/or therapeutic effects for allergic asthma via the inhibition of cytokines, chemokines and chemokine receptors associated with allergic inflammation.

## 1. Introduction

In recent years, the incidence of allergic diseases has been increasing due to environmental pollution, dietary changes, increased exposure to harmful substances and other factors, which are primarily due to rapid industrialization and urbanization. Asthma is a representative allergic disease that affects approximately 300 million people worldwide, and it has become a global health problem as its social and economic burdens have risen with prevalence in recent years [1].

Asthma is a complex chronic lung disease characterized by airway hypersensitivity (AHR), airway inflammation and reversible airway obstruction, and its main pathophysiology is associated with the immune response of type 2 helper T (Th2) cells [2]. In airway inflammation, Th2 cell-derived cytokines such as interleukin (IL)-4, IL-5 and IL-13 play an important role against allergic antigens in regulating and amplifying inflammation. IL-4 induces the generation of immunoglobulin E (IgE) in B lymphocytes and inflammatory cytokines, such as IL-5 and IL-13 [3,4]. IL-5 is responsible for differentiating immature eosinophils into mature cells and increasing their intravascular release and survival [5,6], and IL-13 induces IgE production in B cells and is involved in eosinophilic airway inflammation [7]. Additionally, IL-4 and IL-13 play an important role in causing airway tissue inflammation, mucus hypersecretion, bronchial fibrosis, metaplasia of goblet cells, and smooth muscle cell proliferation [8,9]. Therefore, the influx and activation of Th2 cells into the airways is a key cause of asthma, and suppressing and/or controlling Th2 cells will be effective in preventing and treating asthma.

Recent studies have actively explored natural substances that can suppress and control Th2 cytokines. 18β-Glycyrrhetinic acid, which is the major bioactive component of Glycyrrhizae Radix, strongly suppressed airway hyperresponsiveness, the accumulation of inflammatory cells and levels of IL-5 and IL-13 in bronchoalveolar lavage fluid (BALF) through inhibition of the orphan receptor γ t (RORγt), signal transducer and activator of transcription 6 (STAT6), GATA-binding protein 3 (GATA-3) pathways and upregulation of the forkhead box p3 (Foxp3) transcription pathway [10]. Additionally, cicadae periostracum and oleic acid inhibited inflammatory cell infiltration, AHR and the production of IL-7 and IL-5 in the BALF and OVA-specific IgE in the serum through the inhibition of Th2/GATA-3 and IL-17/RORγt signaling pathways [11]. These results suggest that natural substances can improve allergic asthma and may serve as novel therapeutic components for the treatment of allergic asthma.

Phellodendri cortex (PC), the dried trunk bark of *Phellodendron amurense* RUPR which belongs to the rutaceae family and is native to China, Korea, Japan, Vietnam and eastern Russia, has traditionally been used to treat patients who suffer from gastroenteritis, abdominal pain or diarrhea [12]. PC is widely distributed in its native regions and has diverse geographical sources, which have different ecological environments and other factors that could possibly result in variations in their chemical constituents [13]. The main chemical components of PC include isoquinoline alkaloids, alkaloids, phenolic compounds, phytosterols, etc., and alkaloids such as berberine, phellodendrine, jatrorrhizine and palmatine are known as major bioactive compounds [14].

According to a previous study, the methanol extract of PC attenuated an LPS-stimulated increase in the production of tumor necrosis factor (TNF-α), IL-1β and nitric oxide (NO) in BV2 cells (a mouse microglial cell line) as well as in primary mouse microglia. Molecular mechanisms that govern the attenuation of the levels of mRNAs and proteins of these cytokines and inducible nitric oxide synthase (iNOS) revealed that the PC extract inhibited LPS-stimulated phosphorylation of extracellular signal-regulated kinase (ERK) and activation of nuclear factor κB (NF-κB) [15]. Furthermore, many previous studies revealed that PC has many physiological activities, including anti-microbial [16,17,18], anti-ulcer [12], neuroprotective effects [19] and anti-cancer [20], as well as anti-inflammatory activities [15,21,22]. Although PC is an effective anti-inflammatory natural substance that inhibits the inflammatory response, the effect of PC on allergic asthma has not yet been investigated. Thus, in this study, we investigated the effects of PC on the major pathophysiology of asthma in an ovalbumin-induced mouse asthma model.

## 2. Results and Discussion

### 2.1. HPLC Analysis of PC Main Chemical Components

Alkaloids (berberine, phellodendrine, jatrorrhizine and palmatine, etc.) and limonoids (limonin and obakunone) play an important role in the anti-inflammatory effects of PC. Berberine has been reported for its anti-inflammatory effects by inhibiting basal and 12-O-tetradecanoylphorbol-13-acetate (TPA)-mediated prostaglandin E2 (PGE2) level and cyclooxygenase-2 (COX-2) expression through inhibiting activator protein 1 (AP-1) binding [23], suppressing the functions and differentiation of pro-inflammatory Th1 and Th17 cells, and decreasing Th cell-mediated inflammation through modulating or suppressing other cells assisting autoreactive inflammation [24]. Additionally, limonoids (limonin and obakunone) efficiently reduced NO production and iNOS gene expression via an NF-κB-mediated pathway, a key transcription factor involved in inflammation [25]. Based on this background, we conducted an HPLC analysis to determine the content of alkaloids (Phellodendrine, Jatrorrhizine, Palmatine and Berberine) and limonin in PC before the experimental materials were used.

**Figure 1:**
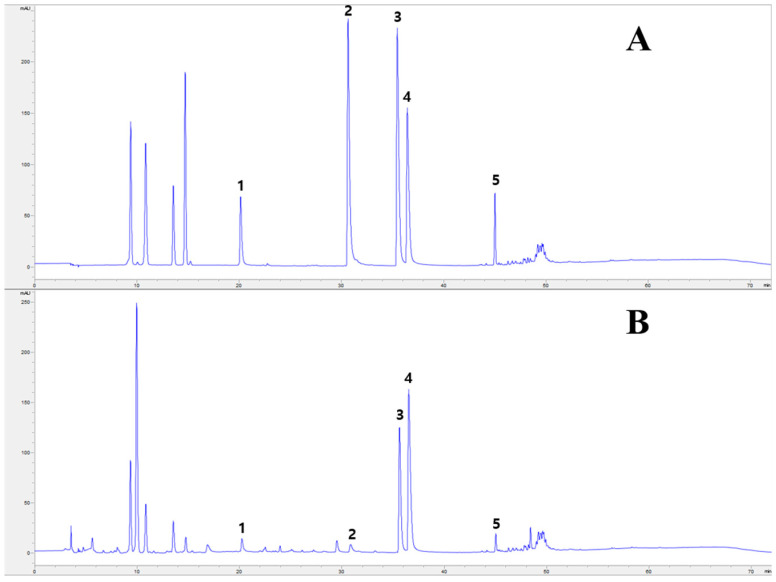
Chromatograms for chemical analysis by HPLC. (**A**) Standard mixture. Peak identities: phellodendrine (1), jatrorrhizine (2), palmatine (3), berberine (4), limonin (5). (**B**) Phellodendri Cortex (PC) sample.

Chromatograms of the standard mixture are shown in Figure 1A. Four alkaloids (phellodendrine, jatrorrhizine, palmatine and berberine) and limonin were identified by the HPLC-diode array detector (DAD) method in the standard mixture. Among the components, berberine (16.09 ± 0.19 mg g^−1^) was present at the highest content followed by palmatine (9.07 ± 0.04 mg g^−1^), limonin (3.90 ± 0.03 mg g^−1^), phellodendrine (3.43 ± 0.21 mg g^−1^) and jatrorrhizine (1.01 ± 0.05 mg g^−1^) (Table 1). Yang et al. [26] reported that the compound limonin (10.31–15.48 mg g^−1^) was present at the highest content followed by berberine (3.72–14.65 mg g^−1^), palmatine (2.21–5.51 mg g^−1^), phellodendrine (1.06–3.54 mg g^−1^) and jatrorrhizine (0.23–0.93 mg g^−1^) among the constituents of 21 PC samples collected from different locations in China. Compared with a previous study, there was a difference in the highest component and its content, but the lowest component was the same as jatrorrhizine. The composition variation may be due to a variety of factors, such as ecological environments, climatic factors, harvest time, drying and storage conditions.

Considering the complexity of herbal medicines, differences in the content of various components may greatly affect the therapeutic effects. Thus, the Korean and Chinese Pharmacopoeia describe the minimal content of berberine (berberine hydrochloride) and palmatine (palmatine hydrochloride) in PC as 0.60% and 0.30% [14,27]. In this study, the contents of berberine and phellodendrine were 1.61% and 0.91%, respectively, which exceeded the minimum quality levels in the Korean and Chinese Pharmacopoeia.

### 2.2. Inhibitory Effects of PC on OVA-Induced AHR and Histological Changes of Lung Tissues in Mice

**Figure 2:**
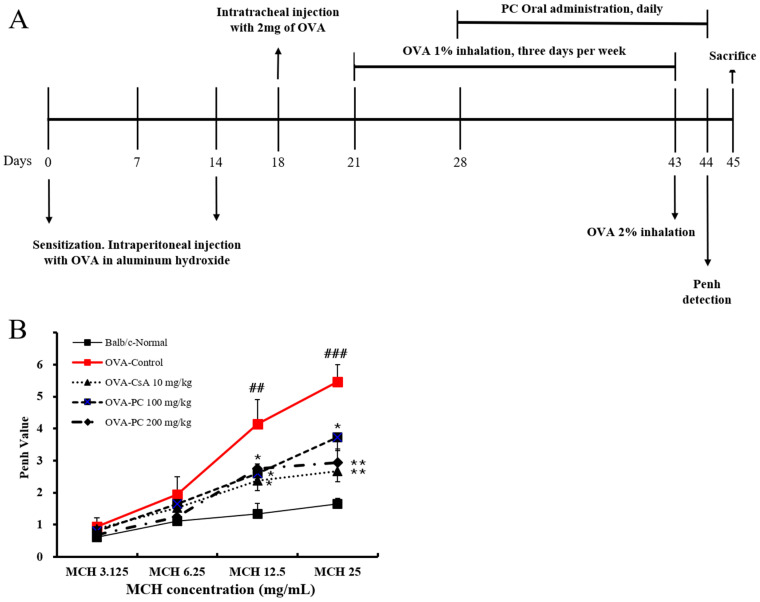
Scheme illustration of ovalbumin sensitization and challenge protocol (**A**). The effect of PC on the airway hyperresponsiveness (AHR) of OVA-induced mice (**B**). Results are expressed as mean ± SEM (n = 4 per group). Statistical analysis of data was performed using Student’s *t*-test. ##: *p* < 0.01 and ###: *p* < 0.001 compared with normal group. *: *p* < 0.05 and **: *p* < 0.01 compared with control group.

AHR is defined by an exaggerated response of the airways to nonspecific stimuli such as histamine, methacholine and cold air, which results in airway obstruction and breathing difficulties caused by excessive narrowing of the pulmonary airways, which is instigated by the shortening of the airway smooth muscle [28]. The degree of AHR is usually in proportion to the severity of the underlying asthma [29]. In the present study, AHR in an OVA-induced mouse asthma model was estimated using the methacholine (MCH) test (enhanced pause (Penh) system). As shown in Figure 2B, the Penh value of the control group was increased by MCH in a dose-dependent manner, and was significantly higher at MCH 12.5 and 25 mg mL^−1^ compared with the normal group. On the other hand, the PC-treated group (100 and 200 mg kg^−1^) significantly reduced MCH-induced AHR, and PC-200 mg kg^−1^ attenuated the increased AHR more effectively than PC-100 mg kg^−1^, showing a similar effect to CsA. The inhibitory effect of PC on AHR may be associated with non-berberine components. According to Jing et al. [30], PC n-butyl alcohol extract (non-berberine) can inhibit L-type Ca2^+^ channels, blocking high K^+^-induced contractions in healthy and asthmatic airway smooth muscle. Additionally, it inhibits transient receptor potential canonical 3 and/or STIM/Orai channels to reduce acetylcholine-induced contractions in both types of airway smooth muscle.

**Figure 3:**
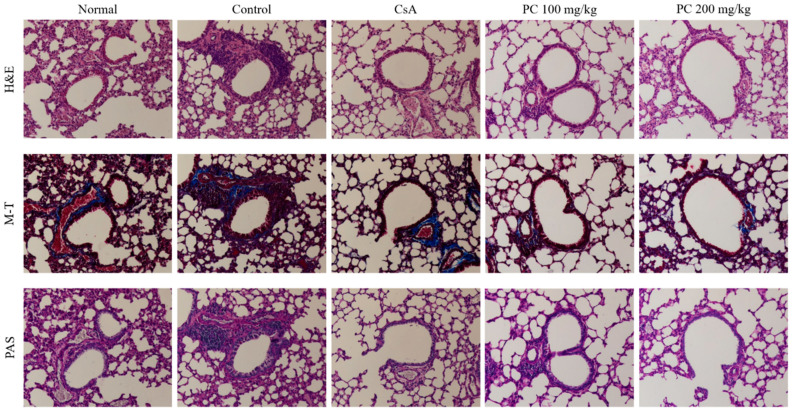
Effects of PC on histology of lung tissue in OVA-induced mice. Results are expressed as mean ± SEM (n = 4 per group). H&E: Hematoxylineosin (×100); M-T: Masson’s trichrome (×100); PAS: periodic acid–Schiff (×100).

One of the major characteristics of asthma is the unique histological and structural changes that are associated with airway inflammatory reactions including hypertrophy and hyperplasia of airway smooth muscle cells, goblet cell hyperplasia and subepithelial fibrosis [31,32]. To investigate the inhibitory effects of PC on these histological changes, lung tissues were stained with hematoxylin and eosin (H&E), Masson’s trichrome (M-T), and periodic acid–Schiff (PAS). As shown in Figure 3, H&E and M-T staining revealed that the control group showed an increased infiltration of various inflammatory cells, such as eosinophils and mast cells, admixed with lymphocytes, as well as collagen deposition compared with the normal group. In contrast, the PC groups showed decreased infiltration of inflammatory cells in lung tissues, as well as less deposition of collagen compared with the control group. Additionally, PAS staining was used to assess goblet cell hyperplasia. Increased goblet cell hyperplasia and mucus secretion were observed in the control group compared with the normal group, while the PC and CsA groups showed a decrease in goblet cell hyperplasia and mucus secretion compared with the control group. These results suggest that PC may attenuate the main pathophysiology of allergic asthma, including histological and structural changes such as airway remodeling, as well as AHR in an OVA-induced mouse asthma model.

### 2.3. Inhibitory Airway Eosinophil Accumulation and Influx of Inflammatory Cells into the Lung and BALF

**Figure 4:**
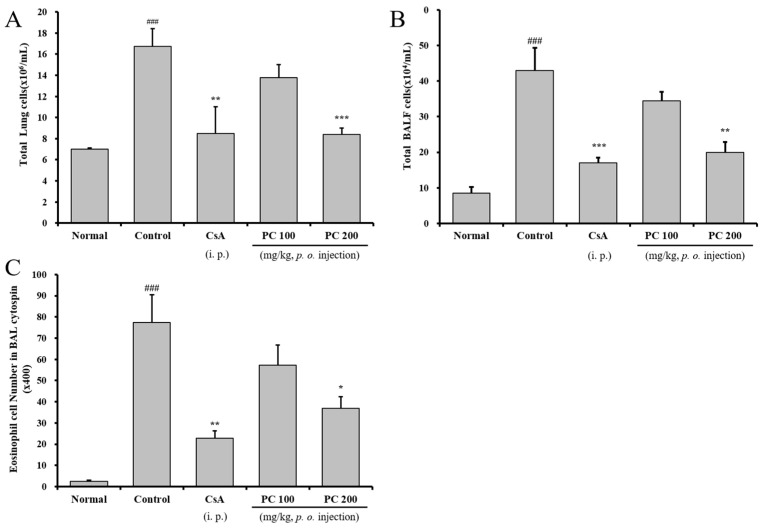
The effects of PC on total lung cells, total BALF cells and eosinophils in BALF. Total lung cells (**A**), total BAL cells (**B**) and eosinophils in BALF (**C**). Results are expressed as mean ± SEM (n = 4 per group). Statistical analysis of data was performed using Student’s *t*-test. ###: *p* < 0.001 compared with normal group. *: *p* < 0.05, **: *p* < 0.01 and ***: *p* < 0.001 compared with control group.

In the case of allergic asthma, inflammatory cells including Th2 cells, eosinophils, monocytes and mast cells increase in the lungs, and lymphocytes that recognize antigens activate in the lymph nodes and migrate to inflammatory tissues [33]. Eosinophils preferentially accumulate at sites of allergic inflammation and contain inflammatory proteins (major basic protein, eosinophil-derived neurotoxin, cationic protein and peroxidase) and leukotrienes, which accelerate direct damage to airway epithelial cells [5,6,34]. To evaluate the effect of PC on the recruitment of cells to the airway, we investigated the cell number of eosinophils in BALF, as well as total lung and BALF cells. As shown in Figure 4, the total number of lung and BALF cells isolated from the control group was 16.8 ± 1.6 × 10^6^ mL^−1^ and 43.0 ± 6.4 × 10^4^ mL^−1^, respectively. The control group showed a significantly higher level than in the normal group (7.0 ± 0.1 × 10^6^ mL^−1^ and 7.0 ± 0.1 × 10^4^ mL^−1^) in total lung and BALF cells. On the other hand, the total number of lung and BALF cells was significantly decreased in PC-200 mg kg^−1^ (8.4 ± 0.6 × 10^6^ mL^−1^ and 20.0 ± 2.8 × 10^4^ mL^−1^) and CsA group (8.5 ± 2.5 × 10^6^ mL^−1^ and 17.0 ± 1.4 × 10^4^ mL^−1^). Additionally, eosinophils in the BALF cytospin of PC-200 mg kg^−1^ and CsA group significantly decreased compared with the control group. PC-100 mg kg^−1^ showed a decreasing trend in eosinophils in BALF, total lung and BALF cells comparable to the control group, although not statistically significant.

Furthermore, to confirm the changes in immune cell subtypes distributions by treatments, lung cells were analyzed by flow cytometry. The absolute numbers of CD4^+^, CD8^+^, CD4^+^CD69^+^, B220^+^CD23^+^, Gr-1^+^CD11b^+^ and CCR3^+^ cells in the lungs of control mice were significantly increased compared with the normal group, but the PC-200 mg kg^−1^ and CsA groups were significantly decreased compared with the control group (Table 2). PC-100 mg kg^−1^ was significantly decreased in CD4^+^, CD4^+^CD69^+^, B220^+^CD23^+^ and Gr-1^+^CD11b^+^ cells; however, its effects were not as prominent as the PC-200 mg kg^−1^ group. CD4^+^ helper T cells are classified based on cytokine production; the cytokines produced by Th2 cells stimulate various cells to trigger the release of IgE and other inflammatory mediators, and CD8^+^ T cells produce cytokines such as IL-4, -5 and -13 [35,36]. In addition, CD69^+^ is known to play an important role in allergic antigen-related eosinophilic airway inflammation and AHR [37,38]. In our study, a reduction in immune cell subtypes including CD11b^+^Gr-1^+^ granulocytes, CCR3^+^ eosinophils, B220^+^CD23^+^ B cells, etc., indicated airway eosinophil accumulation and the influx of inflammatory cells into the lung and BALF in OVA-induced asthma mice. These results support that PC may attenuate histological and structural changes and AHR concerning airway inflammatory reactions and suggest that PC may have preventive and/or therapeutic effects on asthma.

### 2.4. Inhibitory Effects of Th2 Cytokines in BALF and OVA-Specific IgE Production in Serum

As previously described in the Section 1, the main pathophysiology of allergic asthma is associated with the immune response. Th2 cell-derived cytokines such as IL-4, IL-5 and IL-13 against allergic antigens play an important role in regulating and amplifying inflammation, and TNF-α, an inducer of the inflammatory response and a regulator of immunity, is another pleiotropic cytokine produced by several proinflammatory and structural cells known to be crucial in the pathogenesis of chronic inflammatory disorders of the airways [39,40]. Generally, IL-5 differentiates immature eosinophils into mature cells, promotes recruitment to the airway mucosa and prolongs survival in the airway mucosa [41,42]. Eosinophils contain inflammatory proteins and leukotriene, which not only accelerate direct damage to airway epithelial cells but also causes bronchospasm, vascular permeability and edema [6,43]. Furthermore, IL-4 and IL-13 interact directly or indirectly with spatial epithelial cells, fibroblasts, airway smooth muscle cells and endothelial cells to induce airway remodeling, promote iso-switching in IgE secreted from B cells and stimulate IgE production [8,9]. IgE binds to a high-affinity IgE receptor on the mast cell surface and then binds to an allergic antigen to activate the cells, which causes them to release inflammatory mediators such as histamine and leukotrienes that cause airway inflammation and bronchoconstriction [44]. Therefore, blocking TNF-α and controlling the Th2-type immune response may be a therapeutic target in chronic asthma.

**Figure 5:**
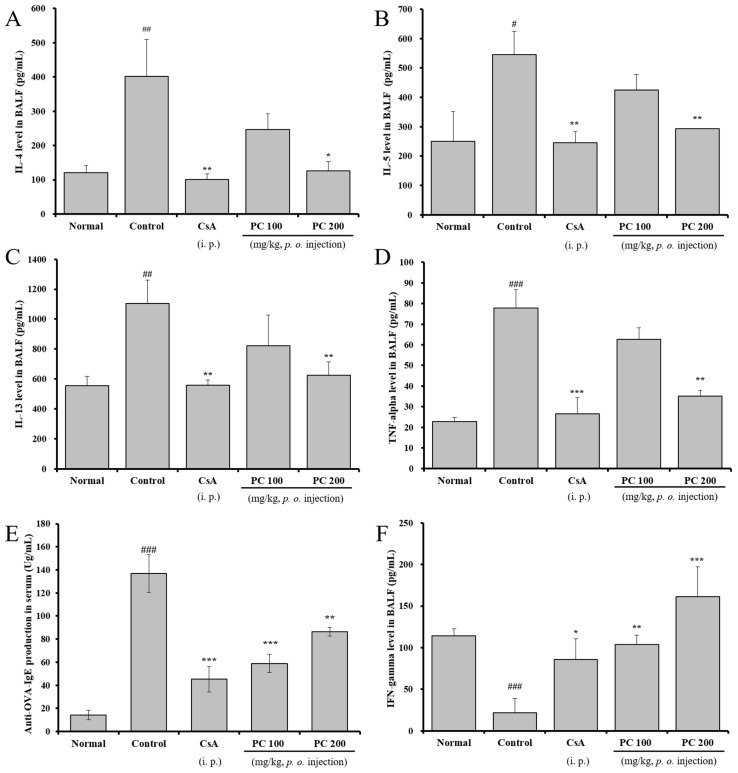
The effects of HD on Th2 cytokines (IL-4, IL-5 and IL-13), TNF-α and IFN-γ in BALF, and the levels of total IgE in serum. IL-4 in BALF (**A**), IL-5 in BALF (**B**), IL-13 in BALF (**C**), TNF-α in BALF (**D**), IgE in serum (**E**) and IFN-γ in BALF (**F**). Results are expressed as mean ± SEM (n = 4 per group). Statistical analysis of data was performed using Student’s *t*-test. #: *p* < 0.05, ##: *p* < 0.01, ###: *p* < 0.001 compared with normal group. *: *p* < 0.05, **: *p* < 0.01 and ***: *p* < 0.001 compared with control group.

In this study, to investigate whether PC affected the production of Th2 cytokines, TNF-α and interferon-γ (IFN-γ) in BALF as well as IgE in serum, their levels were analyzed using ELISA after the final OVA challenge. As shown in Figure 5, IL-4, IL-5, IL-13 and TNF-α production in BALF of the control group was significantly increased compared with the normal group, while PC-200 mg kg^−1^ and CsA groups were significantly decreased compared with the control group. Additionally, IgE production of the PC-100, 200 mg kg^−1^ and CsA groups significantly decreased compared with the control group. Levels in BALF are dependent upon IL-4, IL-5 and IL-13 in OVA-induced asthma mice, and an elevation of IgE levels has been associated with allergic reactions. In contrast, IFN-γ, which is produced by Th1 cells and regulates eosinophil recruitment in the airway by inhibiting Th2 differentiation [45], was significantly increased in PC-100, 200 mg kg^−1^ and CsA groups. These results support that PC suppressed the generation of a Th2-type immune response, several proinflammatory cells and IgE including mast cell activation on the main pathogenesis of asthma.

### 2.5. Effects of PC on mRNA Expression in Lung Tissue

**Figure 6:**
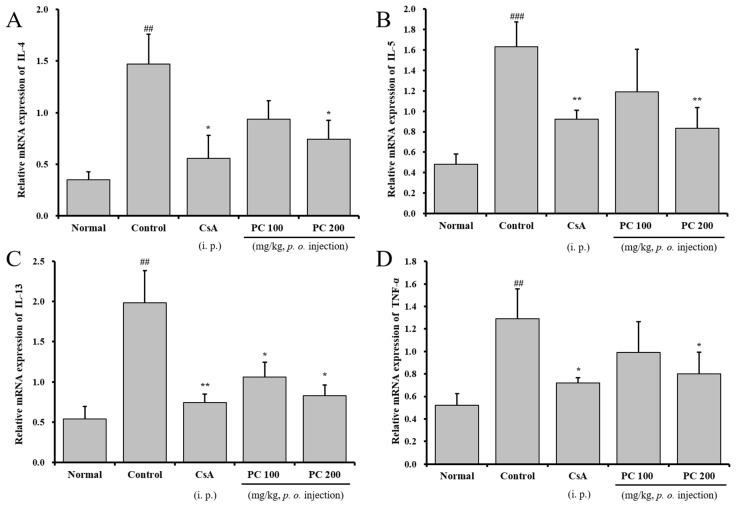

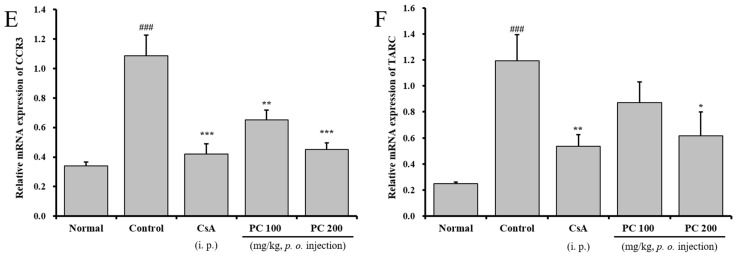
The effects of PC on IL-4, IL-5, IL-13, TNF-α, CCR3 and TARC mRNA expression in lung tissue. (**A**) IL-4 mRNA, (**B**) IL-5 mRNA, (**C**) IL-13 mRNA, (**D**) TNF-α mRNA, (**E**) CCR3 mRNA, and (**F**) TARC mRNA. Results are expressed as mean ± SEM (n = 4 per group). Statistical analysis of data was performed using Student’s *t*-test. ##: *p* < 0.01, ###: *p* < 0.001 compared with normal group. *: *p* < 0.05, **: *p* < 0.01 and ***: *p* < 0.001 compared with control group.

As previously described in the results, PC inhibited Th2 cytokine (IL-5, IL-4 and IL-13) and TNF-α production, airway eosinophil accumulation, and the influx of inflammatory cells and immune cell subtypes in the lung and/or BALF. Further, to demonstrate the inhibitory effects of PC in Th2 cytokine and TNF-α production, we evaluated the relative mRNA expression of asthma-associated cytokines and mediators in lung tissues using qRT-PCR. As shown in Figure 6, IL-4, IL-5, IL-13 and TNF-α transcript expression was significantly upregulated in the lung tissue of the control group. In contrast, PC-200 mg kg^−1^ and CsA groups significantly downregulated transcript expression of Th2 cytokine (IL-5, IL-4 and IL-13) and TNF-α. Moreover, PC-200 mg kg^−1^ significantly downregulated transcript expression of thymus- and activation-regulated chemokine (TARC) and C-C chemokine receptor type 3 (CCR3) in lung tissue. CCR3 is a major chemokine receptor for allergic inflammatory cells such as eosinophils, basophils, mast cells and Th2 cells, and one of the host factors responsible for the selective recruitment of eosinophils to sites of inflammation [46]. Therefore, CCR3 monoclonal antibody can significantly inhibit airway eosinophilia and mucus overproduction. TARC, a lymphocyte-directed C-C chemokine that specifically chemoattracts C-C chemokine receptor 4-positive (CCR4^+^) Th2 cells, is upregulated in allergic inflammation and the specific antibody against TARC attenuates airway eosinophilia and diminishes the degree of airway hyperresponsiveness with a concomitant decrease in Th2 cytokine levels [47]. Therefore, our results demonstrate that PC and CsA decrease IL-4, -5, -13 and TNF-α in BALF through downregulation of the transcript expression of cytokines, TNF-α, CCR3 and TARC in lung tissue and suggest that PC may have preventive and/or therapeutic effects on asthma.

## 3. Materials and Methods

### 3.1. Materials and Reagents

The PC samples were purchased from Donguiherb Co. (Seoul, Republic of Korea), and herbal identification was performed by Professor Heung-Bin Lim. A voucher specimen was deposited in our laboratory. Dried and chopped CP (300 g) was extracted three times with 70% methanol. Then, the extract was filtered under reduced pressure at 40 °C using a vacuum rotary evaporator (BUCHI R-100, Buchi, Switzerland) and dried in a freeze-drier (Lyoph-Pride20, IlShinBioBase Co., Ltd., Dongducheon, Republic of Korea) to yield the CP extract (38.85 g). The yield (*w*/*w*) of the extract was approximately 12.95%. All reagents used in the experiments were of analytical grade and were purchased from Sigma-Aldrich (St. Louis, MO, USA), unless stated otherwise.

### 3.2. Bioactive Compounds Analysis

The four alkaloids (phellodendrine, jatrorrhizine, palmatine and berberine) and limonin of PC were analyzed using a previously described method [13]. Chromatographic analysis was performed on an Agilent 1260 HPLC system (Agilent Technologies, Palo Alto, CA, USA), which consisted of a vacuum degasser, a quaternary pump, an autosampler, a thermostated column compartment and a DAD. Chromatographic data were processed using an Agilent Chemstation. The separation was achieved through the use of a Waters XBridge C18 column (4.6 mm × 250 mm i.d., 5 µm, Waters Corporation, Milford, MA, USA) with a mobile phase composed of (A) 10 mmol NH_4_HCO_3_ in water and (B) ACN. The gradient condition was as follows: 0–13 min, 6–12% B; 13–38 min, 12–40% B; 38–50 min, 40–75% B; 50–55 min, 75–90% B; 55–65 min, 90% B; 65–72 min 90–6% B. Each run was followed by an equilibration period of 8 min with the initial conditions (94% A, 6% B). The injection volume was 5 µL. The flow rate was 0.8 mL/min. The column and autosampler were maintained at 25 °C. The variable wavelength detector was set as follows: 0–13 min, 310 nm; 13–17 min, 275 nm; 17–41 min, 280 nm; 41–55 min, 215 nm.

### 3.3. Animals and Breeding Condition

Six-week-old female BALB mice (20–25 g) were obtained from Daehan Biolink Co. in Seongnam, Republic of Korea. All animals were given solid feed (crude protein ≥ 22.1%, crude fat ≤ 8.0%, crude fiber ≤ 5.0%, crude ash ≤ 8.9%, calcium ≥ 0.6%, and phosphorus ≥ 0.4%) in the form of a standard laboratory diet (Samyang feed, Seoul, Republic of Korea) and tap water ad libitum. The room was maintained at a temperature of 21 ± 2 °C with relative humidity of 50 ± 10%, and a 12 h/12 h light/dark cycle. All animal breeding and experiments were carried out according to the guidelines of the institutional animal care and use committee.

### 3.4. OVA Sensitization and Inhalation

The OVA-induced asthma mouse model method was modified from Lim and Kim [48]. OVA 500 μg/mL (SigmaAldrich, USA) in phosphate buffered saline (PBS) was mixed with an equal volume of 10% (*w*/*v*) aluminum potassium sulfate (SigmaAldrich, USA) in distilled water, incubated for 60 min at room temperature after pH adjustment to 6.5 using 10 N NaOH, and centrifuged at 750× *g* for 5 min. The OVA/alum pellet was re-suspended to the original volume in distilled water. Mice were immunized on 2 different days (on day 0 and on day 14) by an intraperitoneal injection of 0.2 mL alum-precipitated antigen containing 100 µg of OVA bound to 4 mg of aluminum hydroxide in PBS. Four days after the second challenge, an intratracheal injection of OVA 2 mg (on day 18) was administered in the back of the tongue. Starting from the 3rd week (on day 21), OVA solution was administered into the nasal cavity and the respiratory tract using a nebulizer every 30 min for 1 day, 3 days per week, for 22 days (1% OVA in normal saline for first 3 weeks and 2% OVA in normal saline for the last 1 day). One day after the last OVA exposure (2% OVA inhalation), Penh was determined and samples (bronchoalveolar lavage fluid, lung cells and serum) were collected for molecular analyses. The mice were separated into five groups (n = 8 per group) as follows: (1) Normal, (2) OVA-control, (3) OVA-cyclosporine A(CsA) 10 mg kg^−1^, (4) OVA-PC 100 mg kg^−1^, (5) OVA-PC 200 mg kg^−1^; CsA was used as a positive control. The experimental scheme is presented in Figure 1A.

### 3.5. Determination of AHR

AHR in the mice was estimated using the Buxco system (Biosystem XA; Buxco Electronics Inc., Troy, CT, USA). Mice were aerosolized with OVA for 30 min/day, three days/week for three weeks. One day after the final inhalation, mice were given aerosolized saline (0.9% NaCl), followed by increasing doses (3.15, 6.25, 12.5 and 25 mg/mL) of aerosolized methacholine (Sigma-Aldrich). Airway reactivity was then monitored for 30 min, and respiratory curves were converted into Penh values. Penh was calculated as follows:

(1) $$Penh=\frac{PEF}{PIF}\times (\frac{Te-Tr}{Tr})$$

[PEF = Peak Expiratory Flow, PIF = Peak Inspiratory Flow, Te = Expiratory Time, Tr = Relaxation time].

### 3.6. Collection of Blood and BALF

After determination of AHR, the mice were anesthetized by 10% chloral hydrate via intraperitoneal injection, and blood was taken by the cardiac puncture method. Serum was obtained via centrifugation (3000 rpm for 10 min) and stored at −70 °C for use during the experiment. BALF was obtained by injecting 10% fetal bovine serum Dulbecco’s modified Eagle’s medium (DMEM) culture solution at 37 °C into the respiratory tract and then extracting it; this process was repeated 3 times. A hemocytometer (ThermoFisher Scientific, Asheville, NC, USA) was used to investigate the total number of cells in the BALF, which was centrifuged (400× *g* for 4 min) using a Cellspin cytospin centrifuge (Hanil, Incheon, Republic of Korea). BALF cells were placed on glass slides and stained with Diff-Quik to investigate the numbers of eosinophils present among the white blood cells. The BALF supernatant was stored at −70 °C until it was used during the experiment.

### 3.7. Flow Cyrometric Analysis

All antibodies (CD3, CD4, CD8, CD69, CCR3, CD11b, Gr-1 and B220) for flow cytometric analysis were purchased from Becton Dickinson PharMingen (San Diego, CA, USA). Cells from lung tissues and BALF were stained with the indicated antibodies in staining buffer (PBS containing 1% FBS and 0.01% NaN_3_) for 10 min on ice, and analyzed by two-color flow cytometry on a FACScan using CellQuest Pro software(BD Biosciences, Mountain View, CA, USA). The absolute cell number was calculated as a percentage of the total cell number.

### 3.8. Enzyme-Linked Immunosorbent Assay (ELISA)

The levels of cytokines (IFN-γ, IL-4, IL-5 and IL-13) in the BALF and OVA-specific IgE level in serum were measured using ELISA kits (R&D System, Minneapolis, MN, USA). The antibody was diluted in coating buffer; then, 100 μL was added to each microwell and kept overnight at 4 °C. After washing each well 3 times with 200 μL of washing buffer (0.05% Tween 20 in PBS), 100 μL of cell culture supernatant was dispensed. After incubation at room temperature for 1 h and washing twice with washing buffer, a solution (100 μL) of streptavidin-HRP conjugated antibody was added; then, samples were left again for 1 h at room temperature, followed by another wash. To this, 100 μL aliquots of TMB were added and left in darkness for 30 min. Finally, 50 μL of stop solution was added, and the absorbance was measured by the ELISA reader at 450 nm.

### 3.9. Real-Time Quantitative RT-PCR

Total RNA from the lung tissue was extracted using RNAzol B (Tel-Test; Austin, TX, USA) according to the manufacturer’s instructions. cDNA was synthesized from 3 μg of total RNA using a ReverTra Ace-α cDNA Synthesis kit (Toyobo; Osaka, Japan). Gene expression was analyzed with a SYBR Green PCR Master Mix (Applied Biosystems; Grand Island, NY, USA) and 200 nM primers. The PCR was performed as follows: 2 min at 50 °C, 10 min at 94 °C, 40 cycles of 1 min at 94 °C and 1 min at 60 °C. The cycle number at which the emission intensity of the sample rose above baseline was defined as the relative quantity (RQ) and was proportional to the target concentration. RT-PCR was performed using the Applied Biosystems 7500 Fast Real-Time PCR system (Applied Biosystems). Primers and probe sequences are shown in Table 3.

### 3.10. Histology

The lung was removed and immediately fixed in 10% formaldehyde solution, finely cut, and washed for 8 h in running water. After embedding the samples in epoxy, thin sections were cut with a microtome, and stained with hematoxylin and eosin (H&E), Masson’s trichrome stain (M-T) and periodic acid–Schiff (PAS) to confirm inflammatory cell infiltration, collagen deposition and hyperplasia of goblet cells.

### 3.11. Statistical Analysis

Data are expressed as mean ± standard error of the mean (SEM). The results were analyzed using ANOVA or an unpaired Student’s *t*-test followed by Dunnett’s multiple comparison test (SPSS analysis software, version 14.0). Values were considered statistically significant at *p* values of <0.05 (*), <0.01 (**) or <0.001 (***) for the experimental groups compared with the OVA control group comparisons and at *p* values of <0.05 (^#^), <0.01 (^##^) or <0.001 (^###^) for the OVA-induced control group compared with the normal group comparisons.

## 4. Conclusions

This study demonstrated that the oral administration of PC methanol extracts led to significant improvements in the main pathophysiological features of asthma such as airway hypersensitivity (AHR), eosinophil infiltration, serum IgE levels and Th2 cytokine production in an OVA-induced mouse asthma model. Furthermore, PC suppressed the gene expression of cytokines (IL-4, IL-5, IL-13 and TNF-α), chemokine (TARC) and chemokine receptor (CCR3), and attenuated unique histological changes that are associated with airway inflammatory reactions including infiltration of various inflammatory cells, collagen deposition and goblet cell hyperplasia in lung tissue. These results indicate that PC may have preventive and/or therapeutic effects on allergic asthma.

## Figures and Tables

**Table 1:** Quantities of five bioactive markers in PC.

Compound	Content (mg/g)	Correlation Coefficient (R^2^)	Linear Range (µg/mL)	Monitoring Wavelength (nm)
Phellodendrine	3.43 ± 0.21	0.99994	19.92–199.15	280
Jatrorrhizine	1.01 ± 0.05	0.99996	30.76–307.60	280
Palmatine	9.07 ± 0.04	0.99997	24.52–245.17	280
Berberine	16.09 ± 0.19	0.99984	20.49–204.93	280
Limonin	3.90 ± 0.03	0.99997	20.68–206.77	215

Results are expressed as mean ± SEM (n = 5).

**Table 2:** Inhibitory effect of PC on the absolute number of immune cell subtypes in murine OVA-induced asthma lung. Results are expressed as mean ± SEM (n = 4 per group). Statistical analysis of data was performed using Student’s *t*-test. ##: *p* < 0.01, ###: *p* < 0.001 compared with normal group. *: *p* < 0.05, **: *p* < 0.01 and ***: *p* < 0.001 compared with control group.

Cell Phenotypes in Lung	Nomal	OVA-Induced Asthma Mice (Absolute No.)			
		Control	CsA	PC 100 mg kg^−1^	PC 200 mg kg^−1^
CD4^+^ (×10^5^ cells)	20.69 ± 2.76	56.49 ± 2.71 ^###^	19.92 ± 4.22 ***	41.72 ± 5.26 *	33.36 ± 0.27 ***
CD8^+^ (×10^5^ cells)	10.65 ± 0.75	21.32 ± 4.84 ^##^	11.19 ± 3.36 **	18.22 ± 1.28	13.33 ± 0.51 **
Gr-1^+^ (×10^5^ cells)	9.44 ± 0.46	36.71 ± 2.48 ^###^	7.73 ± 1.87 ***	28.34 ± 7.0	6.80 ± 0.95 ***
CCR3^+^ (×10^5^ cells)	5.13 ± 0.01	14.47 ± 1.00 ^###^	4.46 ± 0.68 ***	13.14 ± 0.28	6.53 ± 0.77 ***
CD4^+^CD69^+^ (×10^5^ cells)	1.76 ± 0.03	16.82 ± 3.53 ^###^	3.47 ± 0.65 ***	6.66 ± 0.65 **	6.13 ± 0.05 **
B220^+^ CD3^+^ (×10^5^ cells)	1.56 ± 0.05	27.00 ± 1.09 ^###^	6.84 ± 0.90 ***	17.63 ± 3.49 **	6.84 ± 1.12 ***
Gr-1^+^CD11b^+^ (×10^5^ cells)	5.50 ± 0.69	41.89 ± 3.51 ^###^	9.49 ± 2.31 ***	21.22 ± 2.22 ***	7.80 ± 0.83 ***

**Table 3:** Primer and probe sequence used in real-time PCR analysis.

Gene	Primer	Oligonucleotide Sequence
IL-4	F	5′-GGATGTAACGACAGCCCTCT-3′
	R	5′-GTGTTCCTTGTTGCCGTAAG-3′
IL-5	F	5′-AGCACAGTGGTGAAAGAGACCTT-3′
	R	5′-TCCAATGCATAGCTGGTGATTT-3′
IL-13	F	5′-CAGTTGCAATGCCATCCACA-3′
	R	5′-AGCCACATCCGAGGCCTTT-3′
TNF-	F	5′-ATGAGCACAGAAAGCATGAT-3′
	R	5′-CACACCGACCTTCACCATTTT-3′
CCR3	F	5′-CCCGAACTGTGACTTTTGCT-3′
	R	5′-CCTCTGGATAGCGAGGACTG-3′
TARC	F	5-CATCCATCTCGTGCTACTTGTGTT-3′
	R	5′-CATCTATCCAGTTGGCCTCTGTTT-3′

F, forward; R, reverse.

## Data Availability

The original contributions presented in this study are included in the article. Further inquiries can be directed to the corresponding authors.
